# The Impact of Psychotrauma and Emotional Stress Vulnerability on Physical and Mental Functioning of Patients with Inflammatory Bowel Disease

**DOI:** 10.3390/ijerph20216976

**Published:** 2023-10-25

**Authors:** Boukje Yentl Sundari Nass, Pauline Dibbets, C. Rob Markus

**Affiliations:** 1 Department of Neuropsychology and Psychopharmacology, Faculty of Psychology and Neuroscience, Maastricht University, P.O. Box 616, 6200 MD Maastricht, The Netherlands; 2 Dr. Rath Health Foundation, 6422 RG Heerlen, The Netherlands; 3 Clinical Psychological Science, Maastricht University, P.O. Box 616, 6200 MD Maastricht, The Netherlands

**Keywords:** inflammatory bowel disease, neuroticism, trauma, victimization

## Abstract

Inflammatory bowel disease (IBD) is a chronic health condition thought to be influenced by personal life experiences and emotional stress sensitivity (neuroticism). In the present study, we examined the impact of cumulative trauma experiences and trait neuroticism (as a measure for emotional stress vulnerability) on physical and mental functioning of *n* = 211 patients diagnosed with IBD (112 Crohn’s disease, 99 ulcerative colitis). All patients were assessed for self-reported trauma histories, emotional stress vulnerability, clinical disease activity, functional gastrointestinal (GI) symptoms, and quality of life. Results showed that patients with severe IBD activity have endured significantly more interpersonal trauma and victimization than those with quiescent IBD. Moreover, cumulative trauma was found to exert an indirect (neuroticism-mediated) effect on patients’ symptom complexity, with trauma and neuroticism conjointly explaining 16–21% of the variance in gastrointestinal and 35% of the variance in mental symptoms. Upon correction for condition (using a small group of available controls, *n* = 51), the predictive capacity of trauma and neuroticism increased further, with both predictors now explaining 31% of the somatic—and almost 50% of the mental symptom heterogeneity. In terms of trauma type, victimization (domestic violence and intimate abuse) proved the best predictor of cross-sample symptom variability and the only trauma profile with a consistent direct and indirect (neuroticism-mediated) effect on patients’ mental (QoL) and physical fitness. Results are consistent with the growing body of evidence linking experiential vulnerability factors (trauma and neuroticism) and associated feelings of personal ineffectiveness, helplessness, and uncontrollability to interindividual differences in (GI) disease activity and quality of life.

## 1. Introduction

Inflammatory bowel disease (IBD) is a chronic health condition marked by recurrent episodes of inflammation of the gastrointestinal tract. It includes two subtypes, better known as Crohn’s disease (CD) and ulcerative colitis (UC), distinguished mainly on the basis of the site of inflammation, which in UC is confined to the colon, but in CD may affect the entire gastrointestinal (GI) tract. Both conditions are highly unpredictable in nature, with stress (psychotrauma in particular) playing an important exacerbating role, accounting for a significant proportion of patients’ interindividual symptom variability. Specifically, stressful or traumatic experiences are known to activate the brain–gut axis, a complex communication system involving bidirectional interactions between the central nervous system, the autonomic nervous system, the hypothalamic–pituitary–adrenal (HPA) axis, and the digestive tract [1,2,3,4,5], allowing the brain to exert its regulatory influence over the gut, with the additional effect that a person’s GI health cannot be considered in isolation from his/her psychological and intersubjective functioning. Still, gastroenterologists and other health care providers pay surprisingly little attention to the influence of (inter)personal life experiences and/or emotional stress sensitivity on the functioning of IBD patients [6,7].

A first observation indicative of a meaningful relation between traumatizing stress and GI pathology is the high prevalence of psychotrauma and post-traumatic stress disorders (PTSDs) in GI (including IBD) patients. In the 1990s, it was already established that over 40% of GI patients attending a gastroenterological clinic had a history of physical or sexual abuse [8,9,10]. Moreover, those exposed to abuse exhibited significantly more inflammatory and functional bowel symptoms, somatic complaints, physical disability, lifetime surgeries, and a lower quality of life (QoL) than non-abused GI patients [9,11,12,13,14,15]. Likewise, several other traumatic experiences are now increasingly associated with IBD [12,16,17], calling for a further exploration of the relationship between stressful life experiences and GI vulnerability.

A second line of evidence supporting the stress/trauma-GI connection stems from research on the somatic (i.e., GI) correlates of psychotrauma, indicating that adverse life experiences often have profound and long-lasting disruptive effects on GI function. To illustrate, victims of physical or sexual abuse appeared to be more susceptible to a host of GI complaints [18], ranging from chronic abdominal pain, diarrhea, constipation to nausea, vomiting, bloating, and heartburn [19,20,21,22,23]. Likewise, a majority (71%) of battered women exposed to sexual, physical, or psychological partner violence were found to suffer from persistent indigestion, diarrhea, constipation, spastic colon, or pelvic pain [24,25,26,27]. Beyond intimate and domestic violence, GI symptoms and/or illnesses are also highly prevalent in people who experienced war or war-related captivity [28,29,30,31,32], making it all the more likely that negative, traumatic life experiences significantly amplify and/or increase the risk of (exacerbating) GI pathologies.

The apparent relationship between traumatic or stressful experiences and GI pathology might, however, not be a direct one. Whether a person becomes symptomatic in the aftermath of a traumatic experience seems best predicted by the degree of self-efficacy experienced at the time of the event, i.e., the degree to which the individual felt able to manage or control the situation at hand [33,34,35,36]. The greatest GI (inflammatory/functional) symptom aggravation is thus to be expected in the aftermath of traumatic or stressful events relative to which feelings of being ineffective, incompetent, helpless, and unable to function as an effective agent dominated. Indeed, experiences of a lack of mastery and control are often accompanied by detrimental (functional and structural) changes in the GI tract. To illustrate, uncontrollability during stress (e.g., during “helplessness” test paradigms) was found to predispose animals to gastric ulceration [37], intestinal inflammation, intestinal epithelial permeability [38,39,40,41], altered composition of the microbiota [42], hyper-reactivity of the GI tract [43], and (chemically induced) colitis [44]. Similarly, in patients suffering from IBD, episodes of bleeding and relapse were shown to occur almost exclusively following a subjective experience of being “absolutely crushed”, “helpless”, “hopeless”, “powerless”, “shocked”, and “endangered or enraged but powerless to do anything” [45]. Thus, to summarize, uncontrollable stressful experiences may severely increase IBD patients’ inflammatory and functional symptom profiles and decrease their quality of life, most likely as a function of diminished efficacy experiences.

A commonly acknowledged cognitive–emotional predisposition for frequently experiencing stress and uncontrollability due to a poor sense of personal effectiveness is neuroticism. Individuals with high trait neuroticism suffer from a weak sense of agency, heightened sense of incapacity, and a chronically felt inability to manage challenging events and withstand environmental demands, rendering them particularly vulnerable to feelings of helplessness, negatively tinted experiences, and related affective and somatic pathologies [46]. By confirmation, neuroticism is considered a major predictor for the development of PTSD in response to adversity [47] and associated with a wide range of mental and physical disorders. While IBD—too—appears to be more prevalent in individuals with a neurotic disposition [48], the exact influence of neuroticism on patients’ morbidity and trauma-related symptom aggravations remains to be established.

In conclusion, the high prevalence of trauma and emotional stress vulnerability (neuroticism) among IBD patients, the aggravating effect of adverse life events on the clinical expression of GI disease, and the heightened gut sensitivity in victims loaded with interpersonal trauma all point to trauma and felt uncontrollability as important vulnerability factors driving GI (inflammatory and functional) symptomatology. The current study further explored the notion of such experience-driven GI susceptibility in a sample of IBD patients. To this end, we first established the trauma and emotional stress sensitivity (neuroticism) profiles of IBD patients and related them to (interindividual differences in) inflammatory/functional symptom expressions and quality of life (QoL). Here, symptom/QoL scores were expected to fluctuate as a function of past trauma and neuroticism, with neuroticism either mediating or moderating the negative effects of trauma on GI health. I.e., while it is conceivable that trait neuroticism (reflecting feelings of incapacity and uncontrollability) moderates (influences/intensifies) the effect of trauma on (GI) health, it is equally plausible that it decisively determines (mediates) it (i.e., a strong/meaningful effect of psychological trauma on GI disease is only expected in those who experience such trauma as highly severe and uncontrollable). Secondly, the relative influence of trauma type on these relationships was explored, whereby inflammatory and functional bowel symptoms were hypothesized to increase in accord with trauma complexity (degree of threat to the self or other).

## 2. Materials and Methods

### 2.1. Study Sample

A total of *n* = 112 patients diagnosed with Crohn’s disease (CD, 75.9% female, 24.1% male) and *n* = 99 patients diagnosed with ulcerative colitis (UC; 78.8% female, 21.2% male) were recruited via a gastroenterological university clinic (MUMC+), advertisements on social media, local newspapers, and online platforms of patient organizations. Only patients whose self-reports indicated that they had received an official IBD diagnosis were included in the study. For further exploratory analyses, an additional 51 controls without significant GI complaints (66.7% female, 33.3% male) were enrolled using aforementioned recruitment channels complemented with the Maastricht University’s Research Participation System. The study was approved by the Ethics Review Committee Psychology and Neuroscience at Maastricht University (ERCPN-205_14_03_2019) and complied with the Helsinki Declaration. Informed consent was obtained from all participants.

### 2.2. Materials

#### 2.2.1. Cumulative Trauma Exposure

*Life Events Questionnaire*. Exposure to adverse life events or trauma was assessed using the Dutch Life Events Questionnaire [49], a 28-item self-report instrument covering 18 negative life events relating to divorce (parents/self), chronic or serious physical illness (self/parent/sibling/partner/child), death (parent/sibling/partner/child), serious psychological problems (self/parent/sibling/partner/child), suicide attempts (self/parent/sibling/partner/child), violence within family or relationship, alcohol/drug abuse within family or relationship, unwanted pregnancy, exposure to a crime, accident, or sexual and physical abuse. Items (scored: experienced = 1; not experienced = 0) were assessed across three life stages (<age 16, age 16 till one year ago, in the past year), yielding three life-phase-specific scores (range 0–28) and one trauma total score (range 0–84), with higher scores denoting more cumulative trauma. Additionally, individual items were grouped into four trauma profiles labeled *Profile 1 ‘Medical disease relative’* (sum of physical illness and death of resp. father/mother/caregiver, sibling, partner, and child(ren)); *Profile 2 ‘Psychopathology relative’* (sum of psychological complaints and suicide of resp. father/mother/caregiver, sibling, partner, and child(ren); *Profile 3 ‘Psychopathology self’* (sum of psychological problems and suicide attempts of the self); and *Profile 4 ‘Victimization’* (sum of domestic violence, subjection to a crime, serious accident, sexual abuse, and physical abuse). For each trauma profile, a trauma total score (sum of all items per profile, with a score range of 0–24, 0–24, 0–6, and 0–15 for factors 1–4) and three life-stage specific scores (sum of all profile items per life-stage, with a score range of 0–8, 0–8, 0–2, and 0–5 for profile 1–4) were calculated.

*Neuroticism.* Neuroticism or emotional stress vulnerability was measured using the neuroticism scale of the Dutch Personality Inventory (Inadequacy Scale) [50]. The 21 items of the scale were scored on two-point scales (True/False, scored 2/0) and yielded a total score in the range 0–42 (with higher scores signaling higher levels of neuroticism) when totaled. The internal consistency of the Inadequacy Scale was found to be satisfactory in the present study (Cronbach’s alpha: 0.86).

#### 2.2.2. Clinical GI Disease Activity

*Patient-based Simple Clinical Colitis Activity Index (P-SCCAI).* The P-SCCAI is a patient-reported inventory of current (past week) ulcerative colitis (UC) activity [51]. It covers six domains measuring bowel frequency during the day (one item), night (one item), blood in feces (one item), wellbeing (one item), urgency of defecation (three items) and extra-colonic manifestations (items featuring four extracolonic manifestations (uveitis, erythema nodosum, and pyoderma gangrenosum) and three items measuring arthritis; total of six items). A total UC disease activity score (sum of all item scores, range 0–20) >2 was used as validated cutoff point [52,53] denoting active disease (with 3–5 signaling mild; 6–11, moderate; and ≥12, severe disease activity).

*Patient Harvey–Bradshaw Index (P-HBI).* The P-HBI [54] is an 11-item self-reported measure of current (past week) Crohn’s disease (CD) activity, mapping symptom expression across five domains: general wellbeing (one item), abdominal pain (one item), number of daily liquid stools (open question), and extraintestinal manifestations (eight items: arthralgia, uveitis, erythema nodosum, aphthous ulcer, pyoderma gangrenosum, anal fissure, fistula, and abscess). A cross-item sum score of 5 served as (previously validated) cutoff point establishing active disease (with 5–7, mild; 8–16 being moderate; and >16 = severe UC activity) [55,56].

*Irritable bowel syndrome symptom severity score (IBS-SSS).* Presence of functional GI symptoms over the past 10 days was measured using the IBS-SSS [57], a composite score of self-reported abdominal pain (severity and number of days experienced), abdominal distension (bloating, swollen tummy), contentment with bowel habits, and disease-related quality of life. Items were scored on visual analogous scales (range 0–100), culminating in an IBS-SSS score in the range 0–500 (with 75–175 marking mild; 175–300, moderate; and >300, severe symptomatology). Cronbach’s alpha for the IBS-SSS was 0.85.

*Inflammatory Bowel Disease Questionnaire (IBDQ).* Disease-related QoL during the past two weeks was assessed using the IBDQ [58]. The IBDQ is a 32-item self-report index addressing four dimensions of life, namely: social (5 items), emotional (12 items), systemic (5 items), and gastrointestinal function (10 items). Items were scored on 7-point scales, where 1 denoted worst and 7 denoted best functioning. Total IBDQ scores (sum of all item scores) ranged from 32 to 224 (10 to 70; 5 to 35; 12 to 84; and 5 to 35 for the respective subscales) with higher scores signaling a better QoL. Cronbach’s alpha for the IBDQ was 0.96.

### 2.3. Procedure

Patients were approached via the gastroenterological university clinic Maastricht (MUMC+), social media, local newspapers, and online platforms of patient organizations. They were invited to sign up via email, after which they were informed about the study and received a link to the online questionnaire package (Qualtrics^®^). After signing the informed consent, the previously described questionnaires were administered in the following order: the IBS-SSS, IBDQ, P-HBI, P-SCCAI, the Dutch Life Events Questionnaire, and the neuroticism scale. Median completion time was 21.5 min.

### 2.4. Data Analysis

All data were first examined for missing values (SPSS version 26 for Mac, IBM Corp., Armonk, NY, USA). For one patient, the IBS-SSS score was missing and for seven patients, the P-HBI scores were lacking. These patients were excluded from the respective analyses. First, *t*-tests were conducted to verify whether Crohn’s and colitis patients (CD | UC) significantly differ in symptom reporting (P-SCCAI, P-HBI, IBS-SSS, IBDQ scores). Since this was not the case, (*t*s < 1.59, *p*s > 0.11), patient groups were merged for further analyses. Next, bivariate correlations showed that age was not meaningfully related to physical/mental symptom severity (IBDQ, P-HBI, and P-SCCAI scores respectively) (rs < 0.10, *p*s > 0.18), allowing it to be omitted as a factor from further analyses. Additional bivariate correlations established a large co-variability between the two IBD activity index scores (P-SCCAI and P-HBI) (*r*(202)*=* 0.79, *p* < 0.001) allowing them to be pooled into a single composite Crohn’s–colitis activity score (P-SCCAI + P-HBI). One-way ANOVAs were performed to compare cumulative trauma, trauma subtype, and neuroticism scores across different disease activity groups (quiescent, mild, moderate, and severe IBD activity). Hierarchical multiple regression analyses were conducted to examine whether neuroticism altered the expected association between trauma exposure (either total trauma or a trauma profile as predictor) and IBD disease activity (i.e., pooled Crohn’s–colitis activity, functional GI symptoms, and disease-related QoL). Here, trauma exposure (Model 1), neuroticism (Model 2) were mean-centered and sequentially added as predictors of changes in disease activity. Significant regressions were further analyzed using Preacher and Hayes PROCESS moderation and mediation analyses (Model 1 and 4, bootstrapping with 5000 resamples). All statistics were evaluated at a (two-tailed) significance level of 5%.

## 3. Results

### 3.1. Sample Characteristics

At study entry, 42.1% of Crohn’s (CD) patients presented with active disease (as indicated by a P-HBI score > 5), and approximately three-quarters (74.1%) reported significant functional GI complaints (IBS-SSS score > 74). As for Colitis (UC) patients, roughly two-thirds (66.7%) suffered active disease (P-SCCAI score > 2), and almost three-quarters (72.4%) experienced functional GI symptoms.

### 3.2. Disease Activity Group Differences in Cumulative Traumatization and Neuroticism

One-way ANOVAs revealed a main effect of disease activity (quiescent, mild, moderate, severe) for total trauma exposure (*F*(3, 203) = 3.83, *p* = 0.01), past victimization (*F*(3, 203) = 3.45, *p* < 0.05), and neuroticism (*F*(3, 203) = 14.58, *p* < 0.001) (see Table 1). Pairwise comparisons using Tukey’s post hoc tests revealed that patients with severe IBD activity experienced significantly more cumulative trauma (*p* < 0.05), interpersonal victimization (*p* < 0.01), and structural feelings of uncontrollability and powerlessness (neuroticism) (*p* < 0.001) than those with quiescent IBD. Moreover, patients with mild (*p* < 0.01) and moderate (*p* < 0.001) disease activity scored significantly higher on the neuroticism scale than those with quiescent disease, while those with mild IBD scored significantly lower than those with severe IBD (*p* < 0.05). The remaining comparisons proved non-significant (*p*s > 0.20).

### 3.3. Correlations between Experiential Vulnerability Factors and Disease Activity

Significant correlations between predictors (total trauma, trauma profiles, and neuroticism scores) and dependent outcome variables (IBS-SSS, Composite CD-UC activity and IBDQ scores) are summarized in Table 2. Both neuroticism and victimization were most closely associated with all outcome measures. Total trauma (TT) and psychopathology of self (PoS) correlated with two of the three disease activity measures (IBD activity score and disease-related QoL, respectively).

### 3.4. Effect of Neuroticism on the Relationship between Trauma and IBD Activity

Hierarchical regression analyses revealed that total trauma (TT) as sole predictor (Model 1) explained a small but significant proportion (3%) of the variance in IBD activity scores (*F*(1, 202) = 6.252, *p* = 0.013, *R*^2^ = 0.03; Table 3). When adding neuroticism as an additional predictor (Model 2) the model more powerfully explained 21% of the variance in IBD activity scores (*F*(2, 201) = 26.512; *p* < 0.001), with TT no longer forming a significant predictor of IBD scores. Additional exploratory analyses with a small group of available controls (*n* = 51) indicated that addition of condition (dummy variable: IBD = 1, controls = 0) as a third predictor (*β* = 0.30; *p* < 0.001) yielded a significant increase in explanatory capacity, with trauma (*β* = 0.05; *p* = 0.35) and neuroticism (*β* = 0.39; *p* < 0.001) now explaining 31% of the variance in IBD activity, *F*(3, 248) = 36.98, *p* < 0.001, *R*^2^ = 0.31.

To further examine and interpret significant regressions, moderation and mediation analyses were performed using the PROCESS macro (Model 1 and 4; bootstrapping with 5000 resamples). Results showed that neuroticism did not moderate (b = 0.022, *p =* 0.14) but, rather, mediated the effect of TT on IBD activity (bootstrapped estimate = 0.27, 95% CI = 0.126, 0.454), with the indirect (neuroticism-mediated) path accounting for 70% of the total effect. Hence, greater trauma accumulation was meaningfully related to a structural experience of incapacity or low controllability and, accordingly, to a greater clinical disease expression. Upon correction for neuroticism, the direct effect of TT on patients’ clinical disease expression ceased to be significant (b = 0.011, *p* = 0.43), reaffirming the indirect influence of TT on patients’ clinical disease profiles.

**Figure 1:**
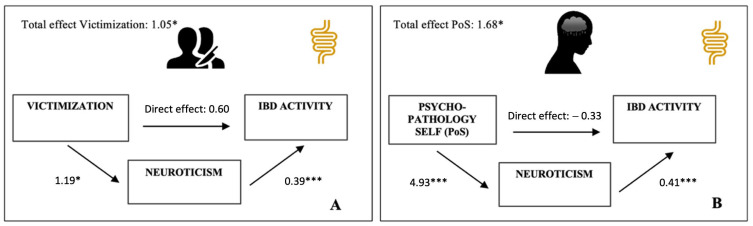
Multiple mediation models of the relationship between traumatic life events and IBD activity. (**A**) Model with victimization predicting IBD activity scores. (**B**) Model with psychopathology of self predicting IBD activity scores. All path estimates are unstandardized regression coefficients. Both trauma profiles are measured on a similar scale, making comparison of the effect estimates straightforward. * *p* < 0.05; *** *p* < 0.001.

To establish which trauma profile was most specific to the association between TT and IBD activity, the two-step regression model was repeated for each trauma profile separately. Out of all traumata, only victimization (Vic) (Model 1: *β* = 0.149; *p* < 0.05) and psychopathology of the self (PoS) (Model 1: *β* = 0.160; *p* < 0.05) were significantly related to IBD activity. Yet, while PROCESS mediation analyses showed that the effect of PoS on clinical disease expression was significantly mediated by neuroticism, (bootstrapped estimate = 2.01, 95% CI = 1.216, 3.003; Figure 1B), the effect of Vic was not (bootstrapped estimate = 0.46, 95% CI = −0.006, 0.987; Figure 1A), even though 43% of Vic’s total effect was accounted for by the indirect (neuroticism-mediated) path.

### 3.5. Effect of Neuroticism on the Relationship between Trauma and Functional GI Symptom Scores

Hierarchical regression analyses indicated that total trauma (TT) as sole predictor (Model 1) could not significantly predict the variance in functional GI symptom scores (FGISS) (*F*(1, 208) = 2.633; *p* = 0.106; *R*^2^ = 0.013; Table 4). Yet, when letting the regression model take into account a possible additional influence of neuroticism (Model 2), it now more powerfully explained 16% of the variance in FGISS (Model 2: *F*(2, 207) = 19.243; *p* < 0.001, *R*^2^ = 0.157). Subsequent exploratory analyses with a small group of available controls (*n* = 51) indicated that inclusion of condition as third predictor (*β* = 0.39; *p* < 0.001) resulted in a further increase in explanatory capacity, with trauma (*β* = 0.01; *p* = 0.93) and neuroticism (*β* = 0.34; *p* < 0.001) now explaining 32% of the FGISS variance, *F*(3, 255) = 40.55, *p* < 0.001, *R*^2^ = 0.32.

**Figure 2:**
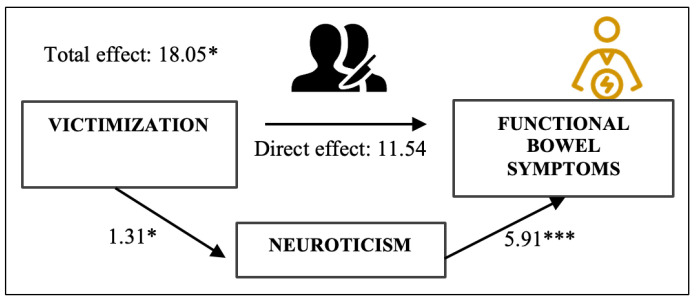
Mediation model of the relationship between victimization and functional bowel symptom severity. All path estimates are unstandardized regression coefficients. * *p* < 0.05. *** *p* < 0.001.

Considering the influence of the individual trauma profiles, victimization (Vic) emerged as the only meaningful predictor of FGISS scores explaining a modest yet significant proportion of interindividual variability in FGISS (Model 1: *F*(1, 208) = 5.851; *p* = 0.016). Hence, where TT had no significant effect on FGISS (Table 3, step 1), Vic did. Subsequent process moderation and mediation analyses confirmed that neuroticism did not moderate, (b = 0.732, *p =* 0.30) but significantly mediated, the association between Vic and FGISS variability (bootstrapped estimate = 6.51; 95% CI: 0.741, 13.553; Figure 2). Upon correction for neuroticism, the direct effect of Vic on patients’ functional bowel symptoms ceased to be significant (b = 11.54, *p* = 0.10), reaffirming the mere indirect influence of Vic on patients’ functional GI complaints.

### 3.6. Effect of Neuroticism on the Relationship between Trauma and Disease-Related QoL Scores

A hierarchical regression analysis including trauma accumulation as sole predictor of interindividual differences in QoL (Model 1) revealed that total trauma (TT) explained a small yet significant proportion (3%) of the variance in QoL (*F*(1, 209) = 6.948, *p* = 0.009; Table 5). When adding neuroticism as second predictor (Model 2), the model now more powerfully explained 35% of the variance in QoL scores (*F*(2, 208) = 56.481; *p* < 0.001, *R*^2^ = 0.352). Subsequent exploratory analyses with a small control group revealed that addition of condition (*β =* −0.33; *p* < 0.001) as a third predictor resulted in another 12% gain in explanatory power, with TT (*β =* −0.02; *p* = 0.62) and neuroticism (*β =* −0.53; *p* < 0.001) now accounting for 47% of variance in QoL. PROCESS moderation and mediation modelling showed that neuroticism did not moderate, (b = 0.003, *p =* 0.96) but mediated the relationship between TT and EIC (bootstrapped estimate = −1.632, 95% CI = −2.506, −0.885). That is, trauma accumulation positively predicted neuroticism (b = 0.74, *p* < 0.001), which, in turn, negatively affected patients’ overall QoL (b = −2.20, *p* < 0.001). After adjustment for neuroticism, TT was no longer meaningfully associated with QoL (b = −0.088, *p* = 0.88), confirming the mere indirect association between TT and QoL.

**Figure 3:**
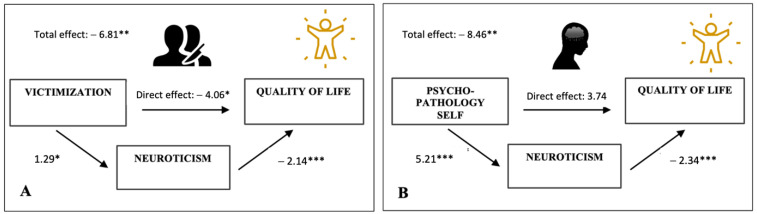
Multiple mediation models of the relationship between different trauma experiences and patients’ overall quality of life (QoL). (**A**) Model with the trauma factor victimization predicting QoL scores. (**B**) Model with trauma factor psychopathology of self predicting QoL scores. * *p* < 0.05; ** *p* < 0.01; *** *p* < 0.001.

Additional regression analyses for the trauma profiles separately identified victimization (*β* = −0.219; *p =* 0.001) as the trauma profile with the most detrimental effect on patients’ QoL (Model 1: *F*(1, 209) = 10.571; *p* = 0.001, *R*^2^ = 0.048), followed by psychopathology of the self (PoS) (Model 1: *F*(1, 209) = 7.445; *p* = 0.007, *R*^2^ = 0.034). Next, PROCESS mediation analyses revealed that the negative effects of both trauma profiles were mediated by neuroticism (Vic: bootstrapped estimate = −2.747, 95% CI = −5.524, −0.375; Figure 3A; PoS: bootstrapped estimate = −12.198, 95% CI = −17.216, −8.170; Figure 3B). However, while TT and PoS merely exerted an indirect (neuroticism-mediated) effect on QoL, Vic influenced QoL both directly (b = −4.06, *p* < 0.05) and indirectly (via neuroticism), hinting at the mere partial mediating role of neuroticism in the association between victimization and overall function.

## 4. Discussion

In the current study, the association between negative life events, emotional stress vulnerability, and symptom variability in patients with inflammatory bowel disease (IBD) was examined. Consistent with a biopsychosocial understanding of IBD [5], the findings provided partial support for the hypotheses that patients’ (inflammatory and functional) symptom presentations fluctuate in accord with their cumulative trauma experiences and emotional stress sensitivity.

First, we found that patients with severe IBD activity have endured significantly more interpersonal trauma and victimization than those with quiescent IBD. Moreover, we established that patients’ cumulative trauma scores were meaningfully associated with (i.e., predictive of) their IBD activity and quality of life scores. That is, the greater the patient’s exposure to interpersonal trauma, the greater their inflammatory symptom expression and the lower their overall quality of life. As anticipated, the effect of trauma on disease activity was mediated by neuroticism, with both factors conjointly accounting for 21% of the variance in inflammatory (GI) symptoms, 16% of the variance in functional GI symptoms, and 35% of the variance in quality of life. More so, upon correction for neuroticism, cumulative trauma lost its explanatory capacity, indicating that structural experiences of uncontrollability and incapacity were at the root of the trauma-induced disease exacerbations. This substantiates existing research suggesting that the symptomatic consequences of trauma are best predicted by the magnitude of felt helplessness, incapacity, or lack of agency. Moreover, it supports data linking structural experiences of uncontrollability and incapacity (neuroticism) to somatic and affective symptomatology [59,60,61] and subjective dysfunction in IBD patients [62]. Exploratory regressions with a small group of available controls (*n* = 51) suggest that the effect of trauma and neuroticism is more pronounced in IBD patients. That is, when adjusting for condition (IBD diagnosis), the explanatory power of trauma and neuroticism in relation to psychological and somatic dysfunction doubled, with the two factors now accounting for 31% of the variance in inflammatory (GI) symptoms, 32% of the variance in functional GI symptoms, and up to 47% of the cross-sample mental (QoL) symptom variance.

Out of all traumata analyzed, victimization (esp. domestic violence and intimate abuse) emerged as the best predictor of the interindividual symptom variability and the only trauma profile with a consistent influence on both mental (QoL) and physical fitness of IBD patients—be it with respect to functional bowel symptoms only by mediation of neuroticism. This is consistent with existing literature [63]. First, it confirms that complex trauma (i.e., pervasive domination of the other over the self) is most disruptive to a person’s self-regulatory capacity. Second, it affirms that individuals with a stronger sense of uncontrollability are more susceptible to the functional GI consequences of victimization. Third, it illustrates that repetitive trauma (as in domestic violence) tends to amplify and generalize the physiologic and affective symptoms of post-traumatic stress [63], with symptoms increasing progressively in relation to stressor intrusiveness. Finally, it illustrates that the traumas that pose the greatest risk in terms of attachment insecurity [64], have the strongest GI symptom-promoting effect—which is coherent with literature linking insecure attachment to heightened affective, neuroendocrine, and inflammatory reactivity [65,66,67,68,69] and studies reporting on the high prevalence of insecure attachment amongst IBD patients [70,71,72].

Interestingly, neuroticism only partially mediated the association between victimization and self-reported wellbeing. This may well be attributed to the fact that the detrimental effect of victimization is supposedly less dependent on—and yet itself dramatically disruptive to—patients’ structurally felt effectiveness and adaptive abilities. As for the remaining trauma profiles, only psychopathology of the self (PoS; patients’ current and past mental comorbidity) coincided with greater physical (inflammation-related) and mental (QoL) function impairment. Yet, upon further analysis, the symptom-aggravating effect of PoS proved merely of an indirect nature, i.e., informed by its inter-relatedness with neuroticism.

To summarize, other than victimization, trauma histories merely exhibited an indirect, neuroticism-mediated effect on patients’ symptom profiles. Hence, neuroticism (reflecting feelings of incompetence and uncontrollability) decisively determined—rather than merely influenced/reinforced—the effect of trauma experiences on (GI) health, implying that a significant effect of psychological trauma on GI illness is mainly to be expected among those who experience such trauma as very severe and uncontrollable. This stands in contrast to previous studies linking cumulative trauma to IBD [12]. Yet, it affirms that—as far as experience-driven (IBD) symptom complexity is concerned—the emphasis is not on trauma accumulation in general, but rather on the experiential state of helplessness, incapacity, and paralysis potentially evoked by it, the manifestation thereof and resistance to which varies as a function of emotional stress vulnerability (neuroticism) and trauma complexity. That is, while feelings of uncontrollability are at the core of neuroticism, they are also widespread in victims of complex trauma (victimization), permanently lowering their thresholds for stress [63].

Current findings must be evaluated considering several limitations. First, trauma histories were evaluated retrospectively and are therefore subject to recall bias. Moreover, trauma scopes were narrowed to a limited number of mainly interpersonal stress experiences with a well-established disruptive effect on the stress-adaptation systems involved in IBD. As such, the study does not contribute to a better understanding of the sensitizing effects of nonsocial experiences, nor of the synergetic effect of social and nonsocial stressors. Third, given the non-specificity of the GI symptomatic repertoire and absence of a sufficiently sensitive (bio)marker of inflammatory activity, the reading of IBD patients’ (inflammatory and functional) symptom profiles was hampered. To resolve these issues in future studies, subjective disease activity indices may be complemented with biochemical markers such as fecal calprotectin that allow for a better differentiation between ongoing IBD activity and concomitant IBS. Fourth, as COVID-19-related measures abruptly restricted patient recruitment in university clinics, we were forced to continue the recruitment procedure online and rely on self-reported IBD diagnoses only, devoid of information on diagnostic procedures followed and parameters used. However, previous research has shown that IBD patients are perfectly capable of providing accurate information (through online self-reports) on their medical history and type of disease [73]. Additionally, considering existing literature identifying inflammation as a risk factor for PTSD, it seems plausible that the causal pathways postulated here are, in fact, bidirectional in nature [74]. To further explore this possibility, inverse mediation models were run (with IBD measures as predictors, neuroticism as mediator, and total trauma as dependent variable), revealing that IBD activity, by augmenting individuals’ stress sensitivity, renders them more vulnerable to structural trauma experiences. Altogether, the findings thus point to a reciprocal relationship between trauma and IBD, with both factors prospectively increasing individuals’ stress sensitivity and therewith their vulnerability to chronic stress experiences and disease progression. Yet, given the cross-sectional nature of the present study, longitudinal studies are needed to better identify the temporal relationships between trauma and IBD activity. Lastly, the results may have been colored by the presence of psychiatric disorders (observed in six patients, one control) as well as the use of psychotropic agents (seven patients, two controls). However, when rerunning all analyses without the affected participants, the same mediating effects of neuroticism emerged while the moderation effects remained insignificant).

## 5. Conclusions

Despite the aforementioned limitations, the current study shows that neuroticism and victimization play a significant role in predicting IBD patients’ (mental/somatic) functioning, most likely by giving rise to an affective stance rotating around feelings of uncontrollability and a confusion about the self as agent. By implication, there where feelings of being ineffective, incompetent, not in control, and unable to function as a relatively autonomous agent prevail, a more aggressive (inflammatory/functional) disease course is to be expected. As such, the present study adds to the growing body of evidence linking experiential vulnerability to IBD expression. It naturally follows that interventions aimed at restoring a sense of agency, rebalancing an overly externally oriented locus of control, and inviting patients into (new) habit formation wherein feelings of mastery, efficacy, choice, and capacity for deliberate action have a natural place are most promising in dampening experientially driven symptom exacerbations in IBD patients.

## Figures and Tables

**Table 1:** Mean difference in trauma exposure amongst different disease activity groups.

	Disease Activity Groups			
	Quiescent (*n* = 84)	Mild (*n* = 65)	Moderate (*n* = 47)	Severe (*n* = 11)
Trauma Type	Mean ± S.D.	Mean ± S.D.	Mean ± S.D.	Mean ± S.D.
Total trauma	3.69 ± 3.17	4.52 ± 3.66	5.11 ± 3.72	7.18 ± 5.83
Victimization	0.48 ± 0.86	0.60 ± 1.14	0.64 ± 1.05	1.64 ± 2.50
Neuroticism	11.33 ± 7.91	16.52 ± 9.63	19.74 ± 8.16	24.36 ± 9.16

**Table 2:** Correlations between traumatic life events (trauma total scores and trauma profiles) or neuroticism and IBD activity.

*n* = 211	IBS-SSS	IBD Activity	IBDQ
Total trauma	0.112	0.173 *	−0.179 **
Trauma profile 1 (medical disease relative)	−0.018	0.060	0.001
Trauma profile 2 (psychopathology relative)	0.042	0.055	−0.068
Trauma profile 3 (psychopathology self)	0.099	0.160 *	−0.185 **
Trauma profile 4 (victimization)	0.165 *	0.149 *	−0.219 ***
Neuroticism	0.396 ***	0.454 ***	−0.593 ***

IBS-SSS = index of functional gastrointestinal (GI) symptoms. IBD activity = patient Harvey–Bradshaw Index score; + patient-based Simple Clinical Colitis Activity Index score. IBDQ = disease-related quality of life (QoL). *, *p* ≤ 0.05; **, p ≤ 0.01; ***, p ≤ 0.001.

**Table 3:** Hierarchical regression analyses testing effects of trauma (total) and neuroticism on IBD activity (composite CD-UC activity scores).

	Step 1			Step 2		
Predictor variables	b	*t*	*β*	b	*t*	*β*
Trauma	0.380	2.500	0.173 *	0.114	0.793	0.052
Neuroticism				0.384	6.738	0.440 ***
*R*^2^ (Δ*R*^2^)	0.030 *			0.209 (0.179) **		
*F change*	6.252 *				45.398 ***	

b, unstandardized regression coefficient; *β*, standardized regression coefficient; *t*, obtained *t*-value; *p*, probability; *R*^2^, proportion of variance explained; Δ*R*^2^, change in proportion variance; *, *p* ≤ 0.05; **, *p* ≤ 0.01; ***, *p* ≤ 0.001.

**Table 4:** Hierarchical regression analyses testing effects of total trauma and neuroticism on functional GI symptoms.

	Step 1			Step 2		
Predictor variables	b	*t*	*β*	b	*t*	*β*
Trauma	3.769	1.623	0.112	−0.119	−0.053	0.958
Neuroticism				5.215	5.951	0.397 ***
*R*^2^ (Δ*R*^2^)	0.013			0.157 (0.144) ***		
*F change*	2.633				35.417 ***	

b, unstandardized regression coefficient; *β*, standardized regression coefficient; *t*, obtained *t*-value; *p*, probability; *R*^2^, proportion of variance explained; Δ*R*^2^, change in proportion variance; ***, *p* ≤ 0.001.

**Table 5:** Hierarchical regression analyses testing effects of total trauma and neuroticism on disease-related QoL.

	Step 1			Step 2		
Predictor variables	b	*t*	*β*	b	*t*	*β*
Trauma	−1.720	−2.636	−0.179 **	−0.088	−0.157	−0.009
Neuroticism				−2.203	−10.131	−0.591 ***
*R*^2^ (Δ*R*^2^)	0.032 **			0.352 (0.320) ***		
*F change*	6.948				102.635	

b, unstandardized regression coefficient; *β*, standardized regression coefficient; *t*, obtained *t*-value; *p*, probability; *R*^2^, proportion of variance explained; Δ*R*^2^, change in proportion variance; **, *p* ≤ 0.01; ***, *p* ≤ 0.001.

## Data Availability

The data presented in this study are available on request from the corresponding author.

## References

[1] D’Andrea W., Sharma R., Zelechoski A., Spinazzola J. (2011). Physical Health Problems after Single Trauma Exposure. J. Am. Psychiatr. Nurses Assoc..

[2] McCrory C., Dooley C., Layte R., Kenny R. (2015). The Lasting Legacy of Childhood Adversity for Disease Risk in Later Life. Health Psychol..

[3] Campbell R., Sefl T., Ahrens C.E. (2003). The Physical Health Consequences of Rape: Assessing Survivors’ Somatic Symptoms in a Racially Diverse Population. Women’s Stud. Q..

[4] De Sousa J.F.M., Paghdar S., Khan T.M., Patel N.P., Chandrasekaran S., Tsouklidis N. (2022). Stress and Inflammatory Bowel Disease: Clear Mind, Happy Colon. Cureus.

[5] Ballou S., Feingold J.H. (2022). Stress, Resilience, and the Brain-Gut Axis: Why Is Psychogastroenterology Important for all Digestive Disorders?. Gastroenterol. Clin. N. Am..

[6] Craven M.R., Quinton S., Taft T.H. (2019). Inflammatory Bowel Disease Patient Experiences with Psychotherapy in the Community. J. Clin. Psychol. Med. Settings.

[7] Keefer L., Sayuk G., Bratten J., Rahimi R., Jones M.P. (2008). Multicenter study of gastroenterologists’ ability to identify anxiety and depression in a new patient encounter and its impact on diagnosis. J. Clin. Gastroenterol..

[8] Drossman D. (1990). Sexual and Physical Abuse in Women with Functional or Organic Gastrointestinal Disorders. Ann. Intern. Med..

[9] Leserman J., Drossman D., Li Z., Toomey T., Nachman G., Glogau L. (1996). Sexual and Physical Abuse History in Gastroenterology Practice. Psychosom. Med..

[10] Walker E.A., Katon W.J., Roy-Byrne P.P., Jemelka R.P., Russo J. (1993). Histories of Sexual Victimization in Patients with Irritable Bowel Syndrome or Inflammatory Bowel Disease. Am. J. Psychiatry.

[11] Baccini F., Pallotta N., Calabrese E., Pezzotti P., Corazziari E. (2003). Prevalence of Sexual and Physical Abuse and Its Relationship with Symptom Manifestations in Patients with Chronic Organic and Functional Gastrointestinal Disorders. Dig. Liver Dis..

[12] Bednarikova H., Kascakova N., Furstova J., Zelinkova Z., Falt P., Hasto J., Tavel P. (2021). Life Stressors in Patients with Inflammatory Bowel Disease: Comparison with a Population-Based Healthy Control Group in the Czech Republic. Int. J. Environ. Res. Public Health.

[13] Drossman D., Li Z., Leserman J., Toomey T., Hu Y. (1996). Health Status by Gastrointestinal Diagnosis and Abuse History. Gastroenterology.

[14] Caplan R.A., Maunder R.G., Stempak J.M., Silverberg M.S., Hart T.L. (2014). Attachment, childhood abuse, and IBD-related quality of life and disease activity outcomes. Inflamm. Bowel Dis..

[15] Kanuri N., Cassell B., Bruce S.E., White K.S., Gott B.M., Gyawali C.P., Sayuk G.S. (2016). The impact of abuse and mood on bowel symptoms and health-related quality of life in irritable bowel syndrome (IBS). Neurogastroenterol. Motil..

[16] Thomann A., Lis S., Reindl W. (2018). P796 Adverse Childhood Events and Psychiatric Comorbidity in a Single-Centre IBD-Cohort. J. Crohn’s Colitis.

[17] Glynn H., Möller S., Wilding H., Apputhurai P., Moore G., Knowles S. (2021). Prevalence and Impact of Post-Traumatic Stress Disorder in Gastrointestinal Conditions: A Systematic Review. Dig. Dis. Sci..

[18] Leserman J., Drossman D. (2007). Relationship of Abuse History to Functional Gastrointestinal Disorders and Symptoms: Some possible mediating mechanisms. Trauma Violence Abus..

[19] Felitti V. (1991). Long-Term Medical Consequences of Incest, Rape, and Molestation. South. Med. J..

[20] Golding J. (1994). Sexual Assault History and Physical Health in Randomly Selected Los Angeles Women. Health Psychol..

[21] Hulme P. (2000). Symptomatology and Health Care Utilization of Women Primary Care Patients Who Experienced Childhood Sexual Abuse. Child Abus. Negl..

[22] Lechner M.E., Vogel M.E., Garcia-Shelton L.M., Leichter J.L., Steibel K.R. (1993). Self-reported medical problems of adult female survivors of childhood sexual abuse. J. Fam. Pract..

[23] Melchior C., Wilpart K., Midenfjord I., Trindade I.A., Törnblom H., Tack J.F., Simrén M., Van Oudenhove L. (2022). Relationship between Abuse History and Gastrointestinal and Extraintestinal Symptom Severity in Irritable Bowel Syndrome. Psychosom. Med..

[24] Talley N., Fett S., Zinsmeister A., Melton L. (1994). Gastrointestinal Tract Symptoms and Self-Reported Abuse: A Population-Based Study. Gastroenterology.

[25] Perona M., Benasayag R., Perelló A., Santos J., Zárate N., Zárate P., Mearin F. (2005). Prevalence of Functional Gastrointestinal Disorders in Women Who Report Domestic Violence to the Police. Clin. Gastroenterol. Hepatol..

[26] Campbell J., Jones A., Dienemann J., Kub J., Schollenberger J., O’Campo P., Gielen A., Wynne C. (2002). Intimate Partner Violence and Physical Health Consequences. Arch. Intern. Med..

[27] Coker A., Smith P., Bethea L., King M., McKeown R. (2000). Physical Health Consequences of Physical and Psychological Intimate Partner Violence. Arch. Fam. Med..

[28] Wang W., Guo X., Yang Y. (2015). Gastrointestinal Problems in Modern Wars: Clinical Features and Possible Mechanisms. Mil. Med. Res..

[29] Pizarro J., Silver R., Prause J. (2006). Physical and Mental Health Costs of Traumatic War Experiences among Civil War Veterans. Arch. Gen. Psychiatry.

[30] McLeay S., Harvey W., Romaniuk M., Crawford D., Colquhoun D., Young R., Dwyer M., Gibson J., O’Sullivan R., Cooksley G. (2017). Physical Comorbidities of Post-Traumatic Stress Disorder in Australian Vietnam War Veterans. Med. J. Aust..

[31] Goulston K., Dent O., Chapuis P., Chapman G., Smith C., Tait A., Tennant C. (1985). Gastrointestinal Morbidity among World War II Prisoners of War: 40 Years on. Med. J. Aust..

[32] Dimsdale J. (1980). Survivors, Victims, and Perpetrators.

[33] Krystal H. (2015). Integration and Self Healing: Affect, Trauma, Alexithymia.

[34] Le L., Morina N., Schnyder U., Schick M., Bryant R., Nickerson A. (2018). The Effects of Perceived Torture Controllability on Symptom Severity of Posttraumatic Stress, Depression and Anger in Refugees and Asylum Seekers: A Path Analysis. Psychiatry Res..

[35] Roemer L., Orsillo S., Borkovec T., Litz B. (1998). Emotional Response at the Time of a Potentially Traumatizing Event and PTSD Symptomatology. J. Behav. Ther. Exp. Psychiatry.

[36] Salcioglu E., Urhan S., Pirinccioglu T., Aydin S. (2017). Anticipatory Fear and Helplessness Predict PTSD and Depression in Domestic Violence Survivors. Psychol. Trauma Theory Res. Pract. Policy.

[37] Overmier J., Murison R. (2000). Anxiety and Helplessness in the Face of Stress Predisposes, Precipitates, and Sustains Gastric Ulceration. Behav. Brain Res..

[38] Wei L., Li Y., Tang W., Sun Q., Chen L., Wang X., Liu Q., Yu S., Yu S., Liu C. (2019). Chronic Unpredictable Mild Stress In Rats Induces Colonic Inflammation. Front. Physiol..

[39] Lennon E., Maharshak N., Elloumi H., Borst L., Plevy S., Moeser A. (2013). Early Life Stress Triggers Persistent Colonic Barrier Dysfunction and Exacerbates Colitis in Adult IL-10−/− Mice. Inflamm. Bowel Dis..

[40] Söderholm J., Yang P., Ceponis P., Vohra A., Riddell R., Sherman P., Perdue M. (2002). Chronic Stress Induces Mast Cell–Dependent Bacterial Adherence and Initiates Mucosal Inflammation in Rat Intestine. Gastroenterology.

[41] Zheng P., Feng B., Oluwole C., Struiksma S., Chen X., Li P., Tang S., Yang P. (2009). Psychological Stress Induces Eosinophils to Produce Corticotrophin Releasing Hormone in the Intestine. Gut.

[42] Murakami T., Kamada K., Mizushima K., Higashimura Y., Katada K., Uchiyama K., Handa O., Takagi T., Naito Y., Itoh Y. (2017). Changes in Intestinal Motility and Gut Microbiota Composition in a Rat Stress Model. Digestion.

[43] Stam R., Croiset G., Akkermans L., Wiegant V. (1996). Sensitization of The Colonic Response to Novel Stress after Previous Stressful Experience. Am. J. Physiol. Regul. Integr. Comp. Physiol..

[44] Milde A., Sundberg H., RØseth A., Murison R. (2003). Proactive Sensitizing Effects of Acute Stress on Acoustic Startle Responses and Experimentally Induced Colitis in Rats: Relationship to Corticosterone. Stress.

[45] Engel G. (1955). Studies of Ulcerative Colitis. III. The nature of psychologic processes. Am. J. Med..

[46] Satchell L., Kaaronen R., Latzman R. (2021). An Ecological Approach to Personality: Psychological Traits as Drivers and Consequences of Active Perception. Soc. Personal. Psychol. Compass.

[47] Breslau N., Schultz L. (2012). Neuroticism and Post-Traumatic Stress Disorder: A Prospective Investigation. Psychol. Med..

[48] Petruo V., Krauss E., Kleist A., Hardt J., Hake K., Peirano J., Krause T., Ehehalt R., von Arnauld de la Perriére P., Büning J. (2019). Perceived distress, personality characteristics, coping strategies and psychosocial impairments in a national German multicenter cohort of patients with Crohn’s disease and ulcerative colitis. Z. Gastroenterol..

[49] Kraaij V., Garnefski N., de Wilde E., Dijkstra A., Gebhardt W., Maes S., ter Doest L. (2003). Negative Life Events and Depressive Symptoms in Late Adolescence: Bonding and Cognitive Coping as Vulnerability Factors?. J. Youth Adolesc..

[50] Luteijn F., Starren J., Dijk H. (1985). Handleiding Bij De NPV.

[51] Bennebroek Evertsz’ F., Nieuwkerk P., Stokkers P., Ponsioen C., Bockting C., Sanderman R., Sprangers M. (2013). The Patient Simple Clinical Colitis Activity Index (P-SCCAI) Can Detect Ulcerative Colitis (UC) Disease Activity in Remission: A Comparison of The P-SCCAI with Clinician-Based SCCAI and Biological Markers. J. Crohn’s Colitis.

[52] Marín-Jiménez I., Nos P., Domènech E., Riestra S., Gisbert J., Calvet X., Cortés X., Iglesias E., Huguet J., Taxonera C. (2016). Diagnostic Performance of the Simple Clinical Colitis Activity Index Self-Administered Online at Home by Patients with Ulcerative Colitis: CRONICA-UC Study. Am. J. Gastroenterol..

[53] Walsh A., Ghosh A., Brain A., Buchel O., Burger D., Thomas S., White L., Collins G., Keshav S., Travis S. (2014). Comparing Disease Activity Indices in Ulcerative Colitis. J. Crohn’s Colitis.

[54] Bennebroek Evertsz’ F., Hoeks C., Nieuwkerk P., Stokkers P., Ponsioen C., Bockting C., Sanderman R., Sprangers M. (2013). Development of the Patient Harvey Bradshaw Index and a Comparison with a Clinician-Based Harvey Bradshaw Index Assessment of Crohn’s Disease Activity. J. Clin. Gastroenterol..

[55] Best W. (2006). Predicting the Crohn’s Disease Activity Index from the Harvey-Bradshaw Index. Inflamm. Bowel Dis..

[56] Vermeire S., Schreiber S., Sandborn W., Dubois C., Rutgeerts P. (2010). Correlation between the Crohn’s Disease Activity and Harvey–Bradshaw Indices in Assessing Crohn’s Disease Severity. Clin. Gastroenterol. Hepatol..

[57] Francis C.Y., Morris J., Whorwell P.J. (1997). The irritable bowel severity scoring system: A simple method of monitoring irritable bowel syndrome and its progress. Aliment. Pharmacol. Ther..

[58] Guyatt G., Mitchell A., Irvine E., Singer J., Williams N., Goodacre R., Tompkins C. (1989). A New Measure of Health Status for Clinical Trials in Inflammatory Bowel Disease. Gastroenterology.

[59] Barlow D., Sauer-Zavala S., Carl J., Bullis J., Ellard K. (2013). The Nature, Diagnosis, and Treatment of Neuroticism. Clin. Psychol. Sci..

[60] Denovan A., Dagnall N., Lofthouse G. (2018). Neuroticism and Somatic Complaints: Concomitant Effects of Rumination and Worry. Behav. Cogn. Psychother..

[61] Shipley B., Weiss A., Der G., Taylor M., Deary I. (2007). Neuroticism, Extraversion, and Mortality in the UK Health and Lifestyle Survey: A 21-Year Prospective Cohort Study. Psychosom. Med..

[62] Moreno-Jiménez B., López Blanco B., Rodríguez-Muñoz A., Garrosa Hernández E. (2007). The Influence of Personality Factors on Health-Related Quality of Life of Patients with Inflammatory Bowel Disease. J. Psychosom. Res..

[63] Herman J. (1992). Complex PTSD: A Syndrome In Survivors of Prolonged And Repeated Trauma. J. Trauma. Stress.

[64] Erozkan A. (2016). The link between types of attachment and childhood trauma. Univers. J. Educ. Res..

[65] Diamond L.M., Hicks A.M., Otter-Henderson K. (2006). Physiological evidence for repressive coping among avoidantly attached adults. J. Soc. Pers. Relatsh..

[66] Ehrlich K.B. (2019). Attachment and psychoneuroimmunology. Curr Opin Psychol..

[67] Gouin J.P., Glaser R., Loving T.J., Malarkey W.B., Stowell J., Houts C., Kiecolt-Glaser J.K. (2009). Attachment avoidance predicts inflammatory responses to marital conflict. Brain Behav. Immun..

[68] Maunder R.G., Hunter J.J. (2001). Attachment and psychosomatic medicine: Developmental contributions to stress and disease. Psychosom. Med..

[69] Pietromonaco P.R., DeBuse C.J., Powers S.I. (2013). Does Attachment Get Under the Skin? Adult Romantic Attachment and Cortisol Responses to Stress. Curr. Dir. Psychol. Sci..

[70] Agostini A., Rizzello F., Ravegnani G., Gionchetti P., Tambasco R., Straforini G., Ercolani M., Campieri M. (2010). Adult attachment and early parental experiences in patients with Crohn’s disease. Psychosomatics..

[71] Agostini A., Moretti M., Calabrese C., Rizzello F., Gionchetti P., Ercolani M., Campieri M. (2014). Attachment and quality of life in patients with inflammatory bowel disease. Int. J. Color. Dis..

[72] Agostini A., Spuri Fornarini G., Ercolani M., Campieri M. (2016). Attachment and perceived stress in patients with ulcerative colitis, a case-control study. J. Psychiatr. Ment. Health Nurs..

[73] Kelstrup A.M., Juillerat P., Korzenik J. (2014). The accuracy of self-reported medical history: A preliminary analysis of the promise of internet-based research in Inflammatory Bowel Diseases. J. Crohns Colitis.

[74] Hori H., Kim Y. (2019). Inflammation and post-traumatic stress disorder. Psychiatry Clin. Neurosci..

